# **α**-Synuclein Modification in an ALS Animal Model

**DOI:** 10.1155/2013/259381

**Published:** 2013-05-12

**Authors:** Eun Jin Yang, Sun-Mi Choi

**Affiliations:** Department of Medical Research, Korea Institute of Oriental Medicine, 483 Expo-ro, Yuseong-gu, Daejeon 305-811, Republic of Korea

## Abstract

Amyotrophic lateral sclerosis (ALS) is a progressively paralytic neurodegenerative disease that can be caused by mutations in Cu/Zn-superoxide dismutase 1 (SOD1). Transgenic mice that overexpress mutant SOD1 develop paralysis and accumulate aggregates of mutant protein in the brainstem and spinal cord. Bee venom (BV), which is also known as apitoxin, is extracted from honeybees and is commonly used in oriental medicine for the treatment of chronic rheumatoid arthritis and osteoarthritis. The purpose of the present study was to determine whether BV affects misfolded protein aggregates such as alpha-synuclein, which is a known pathological marker in Parkinson disease, and ubiquitin-proteasomal activity in hSOD1^G93A^ mutant mice. BV was bilaterally administered into a 98-day-old hSOD1^G93A^ animal model. We found that BV-treated hSOD1^G93A^ transgenic mice showed reduced detergent-insoluble polymerization and phosphorylation of **α**-synuclein. Furthermore, phosphorylated or nitrated **α**-synuclein was significantly reduced in the spinal cords and brainstems of BV-treated hSOD1^G93A^ mice and reduced proteasomal activity was revealed in the brainstems of BV-treated symptomatic hSOD1^G93A^. From these findings, we suggest that BV treatment attenuates the dysfunction of the ubiquitin-proteasomal system in a symptomatic hSOD1^G93A^ ALS model and may help to slow motor neuron loss caused by misfolded protein aggregates in ALS models.

## 1. Introduction

The neuropathological hallmarks of ALS are significant motor neuron loss, Bunina bodies, and the abnormal accumulation of insoluble ubiquitinated cytoplasmic inclusions in lower motor neurons. The neurodegenerative processes in motor neurons are complex. Although several genetic mutations are involved in motor neuron injury in familial ALS, less is known about the genetic and environmental factors that contribute to sporadic ALS. The etiology of ALS is a complex interplay between multiple pathogenic processes, which include misfolded protein aggregates, TDP-43 abnormalities, increased oxidative stress, mitochondrial dysfunction, ribosomal dysfunction, disturbance of intracellular trafficking, dysfunction of the ubiquitin-proteasomal and autophagic systems, increased glutamate excitotoxicity, and impaired axonal transport [1–6].

Parkinson's disease (PD), one of neurodegenerative movement disorders, is caused by selective degeneration of nigrostriatal dopamine neurons in the substantia nigra pars compacta (SNc). The physiological function of alpha-synuclein (*α*-synuclein), which is abundantly expressed in the central nervous system, is not fully understood. Its enrichment in presynaptic terminals and association with vesicles suggest a role for *α*-synuclein in synaptic dynamics. Synuclein has been extensively studied in the pathogenesis of Parkinson's disease (PD). Aggregation of *α*-synuclein in intracytoplasmic Lewy bodies is a key pathological feature of both sporadic and familial PD. In addition, duplication, triplication, or mutations in the *α*-synuclein gene cause some forms of familial PD [7]. Chung et al. have shown *α*-synuclein immunoreactivity in the brain and spinal cord of hSOD1^G93A^ mice [8].

Bee venom (BV) is an oriental medicine that has been used for the treatment of inflammatory diseases such as rheumatoid arthritis and for the relief of pain [9]. BV consists of many biologically active enzymes, peptides, and biogenic amines, such as melittin (the major active ingredient of bee venom), apamin, adolapin, and mast cell-degranulating peptide [10]. BV has been reported to cause growth arrest or cytotoxic effects in hepatocellular carcinoma cells [11]. In addition, the proliferation of melanoma cells, activity of apoptotic enzymes (bcl-2 and caspase-3) in leukemic cells, and activation of the ERK and Akt signaling pathways in renal cancer cells have all been shown to be regulated and/or suppressed by BV [12]. Furthermore, we demonstrated that BV treatment reduced antineuroinflammatory events and increased the survival of hSOD1^G93A^ mice, an ALS animal model used in previous studies [13]. Moreover, we found an increase of modified *α*-synuclein (phosphorylation or nitration) in the spinal cord and brainstem of symptomatic hSOD1^G93A^ transgenic mice [14].

On the basis of these findings, we investigated BV effects on *α*-synuclein modification in the brainstem and spinal cord of symptomatic hSOD1^G93A^ mice. We found that BV treatment reduced the expression of *α*-synuclein modifications such as nitration and phosphorylation. In addition, BV treatment diminished ubiquitinated *α*-synuclein and recovered proteasomal activity in the spinal cord of hSOD1^G93A^ mice.

Taken together, we suggest that BV treatment could be a useful therapy to reduce cell loss or death caused by protein misfolding in a neurodegenerative disease model.

## 2. Materials and Methods

### 2.1. Animals

All of the mice were handled in accordance with the protocols approved by the Institutional Animal Care and Use Committees of the Korea Institute of Oriental Medicine. Hemizygous hSOD1^G93A^ transgenic B6SJL mice were originally obtained from Jackson Laboratories (Bar Harbor, ME). Transgenic mice were identified by PCR as described previously [15]. All of the mice were maintained in standard housing with free access to water and standard rodent chow purchased from Orient Bio (Orient, Gyeonggi-do, Korea).

### 2.2. Bee Venom Treatment

Bee venom (BV) was purchased from Sigma (St. Louis, MO) and diluted in saline. At a dose of 0.1 *μ*g/g, BV was bilaterally injected (subcutaneously) twice a week into 14-week-old male hSOD1^G93A^ transgenic mice at the Zusanli (ST36) acupoint, as determined by the human acupoint landmark and a mouse anatomical reference [16], which is known to mediate anti-inflammatory effects in an ALS animal [13]. Control animals (hSOD1^G93A^) were bilaterally injected (subcutaneously) with an equal volume of saline at the ST36 acupoint.

### 2.3. Immunoprecipitation and Western Blotting Analysis

After the mice were sacrificed, the brainstem and spinal cord were immediately homogenized in ice-cold RIPA buffer containing 50 mM Tris-HCl pH 7.4, 1% NP-40, 0.1% SDS, and 150 mM NaCl and then centrifuged at 14,000 rpm for 30 min at 4°C. The protein concentration in the supernatant was measured using a BCA kit according to the manufacturer's instructions. For immunoprecipitation (IP), 300 *μ*g of protein lysates were immunoprecipitated using an anti-ubiquitin antibody and 10 *μ*L of protein A agarose beads (Santa Cruz, CA, USA). Immunocomplexes were washed three times with RIPA buffer. Proteins were eluted from the beads by boiling in SDS sample buffer, and samples were analyzed by Western blotting with an *α*-synuclein antibody. For Western blotting analysis, proteins were loaded in a SDS-polyacrylamide gel and then transferred onto nitrocellulose membranes (Whatman, MO, USA). The membranes were blocked with 5% nonfat milk dissolved in TBS buffer (50 mM Tris-HCl, pH 7.6, 150 mM NaCl) for 1 h at 37°C. After blocking, the membranes were incubated with the following primary antibodies overnight at 4°C: *α*-synuclein (1 : 1000), phospho-*α*-synuclein (1 : 2000), nitrated-*α*-synuclein (1 : 1000), and ubiquitin (1 : 2000). After washing with TBST buffer (50 mM Tris-HCl, pH 7.6, 150 mM NaCl, and 0.2% Tween-20), the membranes were incubated with an anti-mouse or anti-rabbit IgG conjugated to horseradish peroxidase for 2 h at 4°C. The blots were then washed with TBST buffer and subsequently visualized using an enhanced chemiluminescence (ECL) method. The protein bands were quantitatively analyzed by Kodak Digital Science 1D software (Kodak Company, CT, USA). 

### 2.4. Preparation of a Sequential Extraction of the Mouse Brain and Spinal Cord

Mouse brainstems and spinal cords were isolated and homogenized in ice-cold high salt buffer (50 mM Tris, pH 7.4, 750 mM NaCl, 10 mM NaF, 5 mM EDTA) with a proteinase inhibitor cocktail solution (Calbiochem, CA, USA). The homogenates were centrifuged at 14,000 rpm for 20 min at 4°C. The resulting supernatants were transferred into a new tube (high salt fraction), while the pellets were resuspended in RIPA buffer (50 mM Tris-HCl pH 7.4, 1% NP-40, 0.1% SDS, and 150 mM NaCl) and centrifuged at 14,000 rpm for 20 min at 4°C. The supernatants from this centrifugation were then transferred into a new tube (RIPA fraction), while the pellets were boiled with 0.5% SDS in PBS and centrifuged at 14,000 rpm for 20 min (0.5% SDS fraction).

### 2.5. Proteasomal Activity Assay

Twenty days after saline or BV injection, the male hSOD1^G93A^ transgenic mice were sacrificed. The brainstem and spinal cord from each mouse were removed and homogenized with RIPA lysis buffer and centrifuged at 14,000 rpm at 4°C for 20 min. Then, the supernatant fraction was collected into a new tube, and the protein concentration was determined using a BCA protein kit (Interchim, Paris, France). The proteasomal activity was measured according to the manufacturer's instructions for the 20S Proteasome Activity Assay kit (Chemicon Inc., CA, USA). The fluorescence of the samples was evaluated with a spectrofluorometer at excitation and emission wavelengths of 370 nm and 430 nm, respectively. Proteasomal activity was determined as an increase in the fluorescence of the reaction products.

### 2.6. Immunofluorescence

In this study, 16-week-old hSOD1^G93A^ mice were sacrificed after anesthetizing with pentobarbital and perfusion with phosphate-buffered saline (PBS). The spinal cord tissue of the hSOD1^G93A^ mice was fixed with 4% paraformaldehyde in PBS at 4°C overnight. The lumbar spinal cord was dissected, transferred into 30% sucrose, and then frozen. The lumbar spinal cord was embedded in OCT compound, a freezing solution, and cut along the transverse plane on a Leica Microtome (Leica, Germany). Free-floating sections were washed three times with PBST and then incubated overnight at 4°C with primary antibodies (*α*-synuclein, 1 : 1000, and ubiquitin, 1 : 2000). The sections were washed three times in PBST and then incubated with Alexa Fluor 488 or 568 conjugated secondary antibodies (Molecular Probes, NY, USA). The ventral horns of the spinal cord sections were then imaged using an Olympus fluorescent microscope (Olympus, Tokyo, Japan) under identical exposure settings.

## 3. Results

### 3.1. BV Treatment Reduces Modified *α*-Synuclein Expression in the Brainstem and Spinal Cord of hSOD1^G93A^ Mice

**Figure 1:**
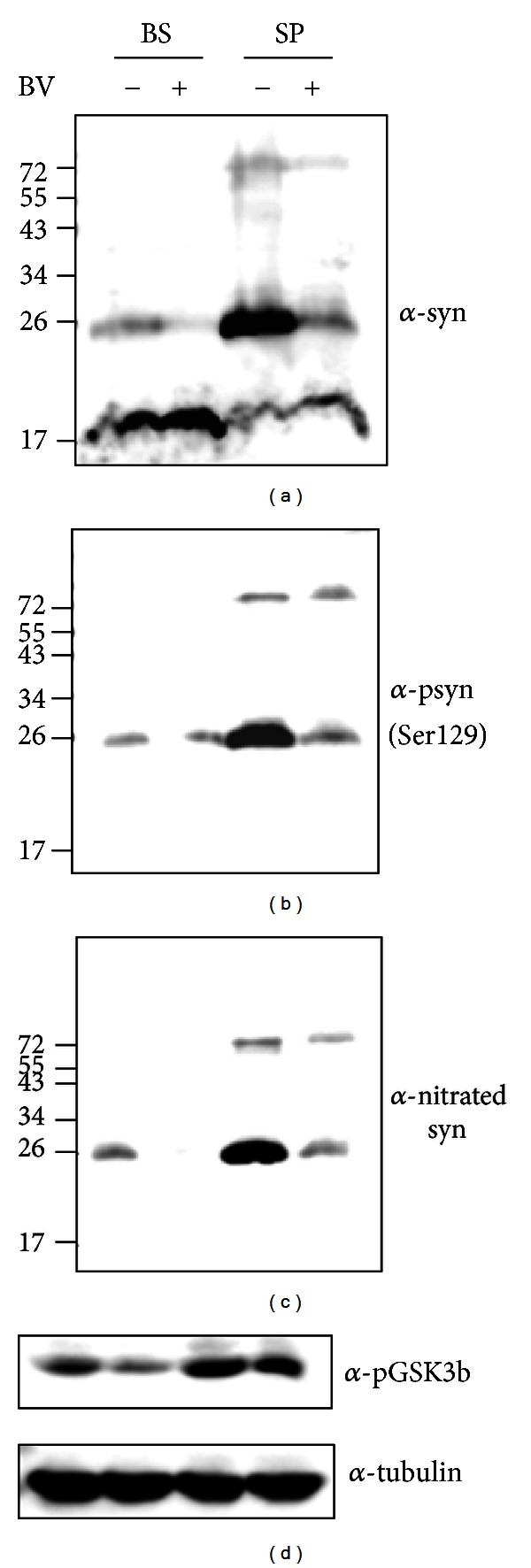
Western blotting analysis of *α-*synuclein expression levels in the spinal cord and brainstem of saline- (*n* = 3) or BV-treated hSOD1^G93A^ mice (*n* = 3). The blot shown is representative of three independent experiments. The total tissue homogenate was immunoblotted with an anti-*α*-synuclein antibody (a), anti-phospho-*α*-synuclein (Ser 129), and anti-nitrated *α*-synuclein. Tubulin served a loading control. BV: bee venom, BS: brainstem, SP: spinal cord.

To assess whether BV treatment affects the expression and modification of *α*-synuclein, the total proteins were extracted in RIPA buffer and loaded onto a 15% SDS-PAGE. Consistent with previous studies, high molecular *α*-synuclein proteins were detected in the saline-treated brainstem and spinal cord (Figure 1(a)). However, phosphorylated or nitrated *α*-synuclein was reduced by BV treatment in the brainstem and lumbar spinal cord of hSOD1^G93A^ mice (Figures 1(b) and 1(c)). Our data suggested that BV treatment attenuates *α*-synuclein modifications caused by motor neuron death in hSOD1^G93A^ mice.

To provide further evidence of increases in phosphorylated *α*-synuclein in symptomatic hSOD1^G93A^ mice, we examined the expression level of phospho-GSK3*β* with RIPA-extracted spinal cord and brainstem tissue in hSOD1^G93A^ mice. As shown in Figure 1(d), the expression of phosphorylated GSK3*β* at Ser 9 was increased in the brainstem and spinal cord of hSOD1^G93A^ mice. Interestingly, BV treatment reduced the phosphorylated levels of GSK3*β* in the brainstem and spinal cord of symptomatic hSOD1^G93A^ transgenic mice compared to age-matched hSOD1^G93A^ mice. This finding suggests that GSK3*β* activation may enhance *α*-synuclein phosphorylation at Ser 129 and contribute to motor neuron loss in symptomatic hSOD1^G93A^ mice. 

### 3.2. Modified *α*-Synuclein Is a Soluble Protein in hSOD1^G93A^ Mice

**Figure 2:**
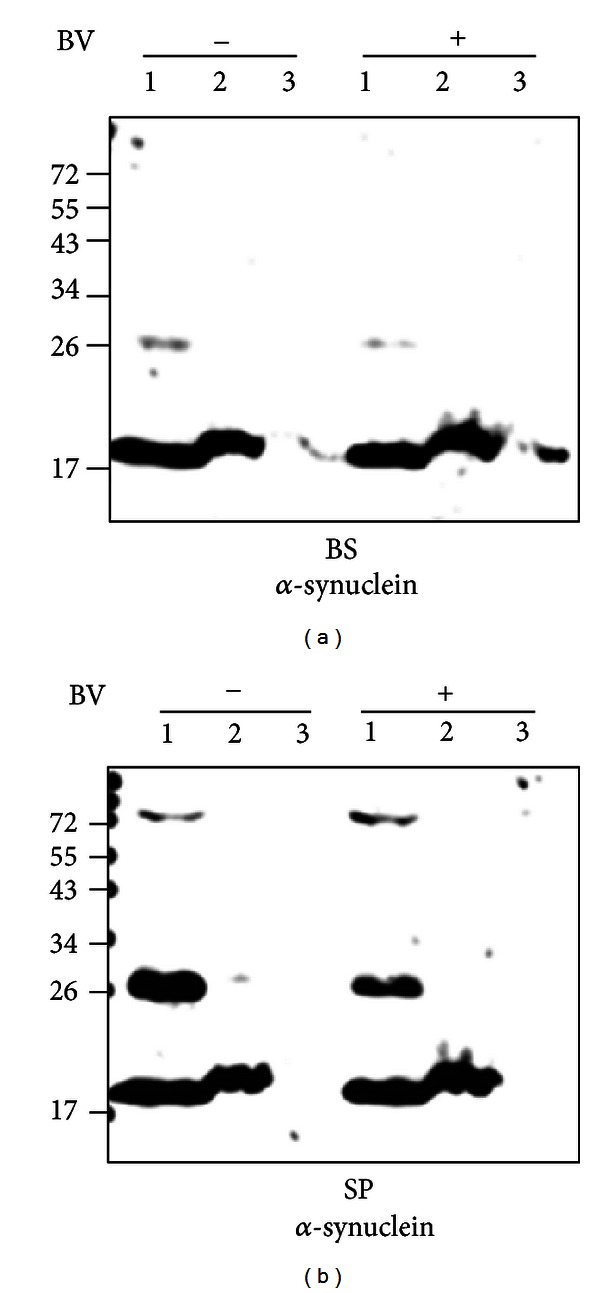
The soluble and insoluble protein fractions were collected from the brainstem (a) or spinal cord (b) of saline- (*n* = 3) or BV-treated hSOD1^G93A^ mice (*n* = 3). Equal amounts of total protein were subjected to a denaturing PAGE as previously described under Section 2, followed by Western blotting analysis with an anti-*α*-synuclein antibody. The blot shown is representative of three independent experiments. 1: high salt buffer, 2: RIPA buffer, 3: 0.5% SDS buffer, BV−: saline-treated hSOD1^G93A^ mice, BV+: BV-treated hSOD1^G93A^ mice.

To determine the solubility of modified *α*-synuclein in the brainstem and spinal cord of hSOD1^G93A^ mice, we examined the solubility of *α*-synuclein with sequentially different buffers (high salt, RIPA buffer and 0.5% SDS buffer as an insoluble buffer). As shown in Figure 2(a), monomeric or modified *α*-synuclein was extracted in high salt buffer as a soluble protein, and BV treatment reduced the expression of modified *α*-synuclein in the brainstem of hSOD1^G93A^ mice. Oligomeric (higher molecular weight) and modified (~26 kDa) *α*-synuclein of the spinal cord in symptomatic hSOD1^G93A^ mice were detected in high salt buffer as a soluble fraction, and modified *α*-synuclein was decreased by BV treatment (Figure 2(b)). However, insoluble *α*-synuclein was not detected in the brainstem or spinal cord of hSOD1^G93A^ mice (Figures 2(a), and 2(b)).

### 3.3. BV Treatment Decreases Ubiquitinated *α*-Synuclein and Rescues the Reduced Proteasomal Activity in Symptomatic hSOD1^G93A^ Mice

However, it has been controversial whether it induces neuronal toxicity. To investigate the effects of BV on ubiquitinated *α*-synuclein, we examined the expression level of ubiquitinated *α*-synuclein in the brainstem and spinal cord of hSOD1^G93A^ mice.

**Figure 3:**
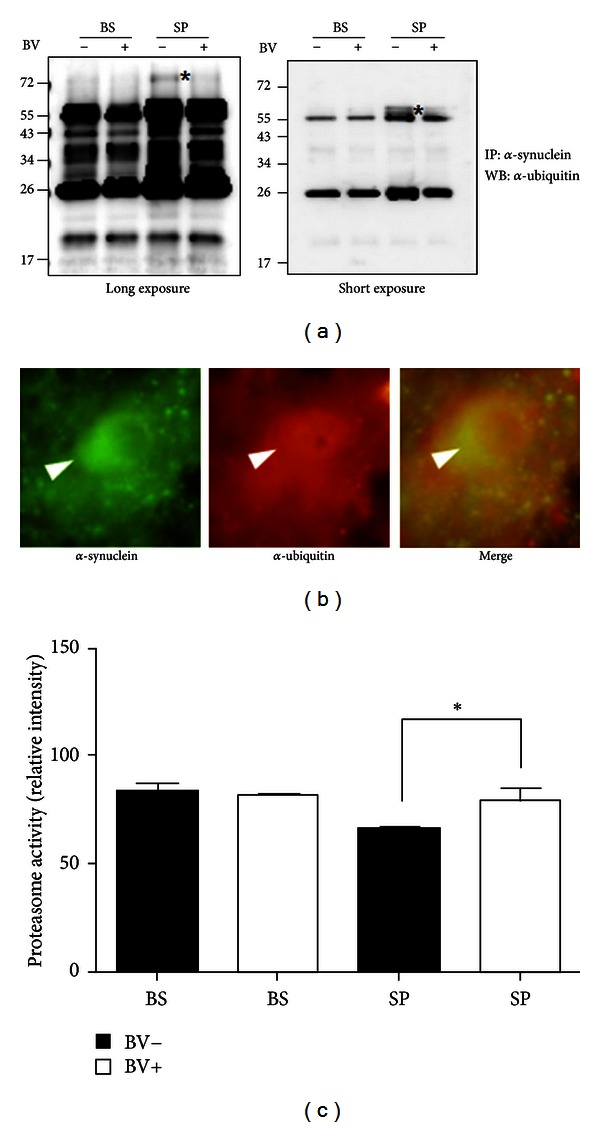
BV treatment attenuates ubiquitinated *α*-synuclein and recovered proteasomal activity in the spinal cord of symptomatic hSOD1^G93A^ mice. The total homogenate was immunoprecipitated with an anti-*α*-synuclein antibody and then immunoblotted with an anti-ubiquitin antibody (a). Ubiquitinated *α*-synuclein of high molecular weight (*) was detected in the spinal cords of saline-treated hSOD1^G93A^ mice, but not in BV-treated mice. Left upper panel is a long-exposure blot and the lower panel is a short-exposure blot. *α*-Synuclein and ubiquitin were colocalized in the lumbar spinal motor neurons of symptomatic hSOD1^G93A^ mice (b). This image is representative of three independent experiments. Proteasomal activity was determined using a fluorescence enzymatic assay (c). BV treatment increased the proteasomal activity 1.5-fold in the spinal cords of symptomatic hSOD1^G93A^ mice (*n* = 4) compared to age-matched controls (*n* = 4). Proteasomal activity was reported in arbitrary fluorescence units (mean ± SEM of three independent experiments). Values that were significantly different from relative controls are indicated with an asterisk when *P* < 0.01. A.F.U. = arbitrary fluorescence units (mean ± SEM).

As shown in Figure 3(a), ubiquitinated *α*-synuclein was reduced by BV treatment in the spinal cord of symptomatic hSOD1^G93A^ mice. In addition, we confirmed that ubiquitinated *α*-synuclein was localized in the motor neurons of hSOD1^G93A^ mouse spinal cords (Figure 3(b)).

Next, to investigate whether modified *α*-synuclein expression affects the protein degradation system, we examined the proteasomal activity in the brainstem and spinal cord of BV-treated or age-matched control hSOD1^G93A^ mice. BV treatment rescued 1.2-fold proteasomal activity in the spinal cord compared to that of age-matched symptomatic hSOD1^G93A^ mice (*P* < 0.05, Figure 3(c)).

## 4. Discussion

In this study, we demonstrated that two subcutaneous injections of 0.1 *μ*g/mL BV resulted in the reduced expression of *α*-synuclein and modified *α*-synuclein (phosphorylation, nitration, or ubiquitination) in the brainstem and spinal cord of symptomatic SOD1^G93A^ transgenic mice. Furthermore, we observed that BV treatment diminished ubiquitinated *α*-synuclein and rescued proteasomal activity in the spinal cords of hSOD1^G93A^ transgenic mice.

Neuroinflammatory responses are induced as a consequence of oxidative and excitotoxic neuronal damage, mitochondrial dysfunction, and protein aggregation [17, 18]. In addition, neuroinflammation may be a direct response to protein aggregation in neurodegenerative diseases [19]. Neuroinflammation and accumulated protein aggregates are related to oxidative stress and mitochondrial dysfunction; accumulated protein aggregates trigger microglial activation and neuroimmune responses [20]. In the case of ALS, abnormal protein aggregation, ubiquitination, and deposition in the CNS are salient features of ALS [21, 22].

The role of neuroinflammation in the disease progression of neurodegenerative diseases, including PD and AD [23] in animal models, has been highlighted in recent studies. For example, in the presence of a preexisting PD-relevant insult, such as 6-OHDA, the loss of parkin or *α*-synuclein overexpression and induction of neuroinflammation synergistically worsen the disease process [24]. Chung et al. have demonstrated that changes in proteins relevant to synaptic transmission and axonal transport, coupled with neuroinflammation, precede *α*-synuclein-mediated neuronal death [25].

Recently, several studies have shown that *α*-synuclein is expressed in symptomatic hSOD1^G93A^ mice [8, 14]. In addition, our previous study demonstrated that *α*-synuclein modification increased in the brainstem and spinal cord of hSOD1^G93A^ transgenic mice compared to age-matched controls [14]. Furthermore, we have shown that treatment with melittin, one of the components of BV, reduced *α*-synuclein modification and increased the activity of proteasomes in the spinal cord of mutant (G93A) hSOD1-expressing mice [14]. This study showed that BV treatment also decreased the expression level of soluble modified *α*-synuclein (phosphorylation or nitration) in the spinal cords of symptomatic hSOD1^G93A^ transgenic mice (Figures 1 and 2). These findings suggest that anti-inflammation by BV treatment may be involved in the restoration of reduced proteasomal activity in symptomatic hSOD1^G93A^ transgenic mice.

Inclusions formed mainly by monoubiquitinated *α*-synuclein are toxic to cells [26]. Baba et al. have shown that the oligomeric or protofibrillar forms are more toxic than the fibrillar forms of *α*-synuclein in disease [27]. In addition, soluble aggregated *α*-synuclein mediates dopaminergic neurotoxicity in *Drosophila* [28]. In an ALS animal model, we found that the level of modified soluble *α*-synuclein (~ 26 kDa) was increased in the brainstem and spinal cord, and BV treatment reduced the modification of *α*-synuclein (Figure 2). Furthermore, we showed that the expression level of ubiquitinated *α*-synuclein was decreased in the spinal cord of symptomatic hSOD1^G93A^ transgenic mice compared to age-matched control mice (Figures 3(a), and 3(b)). This result suggests that BV treatment may attenuate the impairment of proteasomal activity by the expression or accumulation of modified proteins involved in neurodegenerative events. However, further study of whether fibrillar or oligomeric forms *α*-synuclein are more toxic than soluble *α*-synuclein in ALS animal or cell models is needed.

The ubiquitin-proteasome system is a major proteinase system that plays important roles in a vast array of cellular processes, including protein trafficking, antigen presentation, and protein degradation under physiological conditions [29].

Ubiquitinated proteins accumulate to produce protein aggregates in AD, PD, and ALS [30–32]. In the case of ALS, these aggregates are thought to be composed of ubiquitin, SOD-1, some subunits of the proteasome, and neurofilaments [31, 33]. Accumulation of nitrated proteins is also found in the brains of patients with AD, PD, and ALS or in mutant Cu/Zn-superoxide dismutase (SOD-1) or mutant *α*-synuclein transgenic animals [31]. However, there is still no consensus regarding the pathway responsible for *α*-synuclein degradation. It is unclear whether *α*-synuclein degradation depends on the proteasome, chaperone-mediated autophagy, or macroautophagy [34, 35].

Autophagy inhibition promotes the accumulation and subsequent aggregation of monoubiquitinated forms of the *α*-synuclein protein [26]. Recent evidence suggests that autophagy may play a compensatory role when the UPS function is damaged, and vice versa [36]. Inhibition of the UPS has been shown to activate autophagy-mediated protein degradation [37], and suppression of autophagy leads to the accumulation of ubiquitinated proteins in the cytosol [38]. In ALS models, several studies have shown that autophagy dysfunction contributes towards motor neuron loss [39–42]. Thus, further studies are needed to demonstrate whether an increase of *α*-synuclein modification in an ALS animal model is caused by autophagy misregulation.

## 5. Conclusion

This paper demonstrated that BV-treated hSOD1^G93A^ transgenic mice showed reduced detergent-insoluble polymerization and phosphorylation of *α*-synuclein. Furthermore, phosphorylated or nitrated *α*-synuclein was significantly reduced in the spinal cords and brainstems of BV-treated hSOD1^G93A^ mice and reduced proteasomal activity was revealed in the brainstems of BV-treated symptomatic hSOD1^G93A^. From these findings, we suggest that BV treatment attenuates the dysfunction of the ubiquitin-proteasomal system in a symptomatic hSOD1^G93A^ ALS model and may help to slow motor neuron loss caused by misfolded protein aggregates in ALS models.

## References

[1] Shaw PJ (2005). Molecular and cellular pathways of neurodegeneration in motor neurone disease. *Journal of Neurology, Neurosurgery and Psychiatry*.

[2] Kabashi E, Valdmanis PN, Dion P, Rouleau GA (2007). Oxidized/misfolded superoxide dismutase-1: the cause of all amyotrophic lateral sclerosis?. *Annals of Neurology*.

[3] Ström A-L, Gal J, Shi P, Hayward LJ, Zhu H (2008). Retrograde axonal transport and motor neuron disease. *Journal of Neurochemistry*.

[4] Rothstein JD (2009). Current hypotheses for the underlying biology of amyotrophic lateral sclerosis. *Annals of Neurology*.

[5] Lagier-Tourenne C, Cleveland DW (2009). Rethinking ALS: the FUS about TDP-43. *Cell*.

[6] Kanekura K, Suzuki H, Aiso S, Matsuoka M (2009). ER stress and unfolded protein response in amyotrophic lateral sclerosis. *Molecular Neurobiology*.

[7] Singleton AB, Farrer M, Johnson J (2003). alpha-Synuclein locus triplication causes Parkinson's disease. *Science*.

[8] Chung YH, Joo KM, Kim MJ, Cha CI (2003). Immunohistochemical study on the distribution of alpha-synuclein in the central nervous system of transgenic mice expressing a human Cu/Zn superoxide dismutase mutation. *Neuroscience Letters*.

[9] Kwon YB, Lee JD, Lee HJ (2001). Bee venom injection into an acupuncture point reduces arthritis associated edema and nociceptive responses. *Pain*.

[10] Tu WC, Wu CC, Hsieh HL, Chen CY, Hsu SL (2008). Honeybee venom induces calcium-dependent but caspase-independent apoptotic cell death in human melanoma A2058 cells. *Toxicon*.

[11] Li B, Gu W, Zhang C, Huang XQ, Han KQ, Ling CQ (2006). Growth arrest and apoptosis of the human hepatocellular carcinoma cell line Bel-7402 induced by melittin. *Onkologie*.

[12] Moon DO, Park SY, Heo MS (2006). Key regulators in bee venom-induced apoptosis are Bcl-2 and caspase-3 in human leukemic U937 cells through downregulation of ERK and Akt. *International Immunopharmacology*.

[13] Yang EJ, Jiang JH, Lee SM (2010). Bee venom attenuates neuroinflammatory events and extends survival in amyotrophic lateral sclerosis models. *Journal of Neuroinflammation*.

[14] Yang EJ, Kim SH, Yang SC, Lee SM, Choi SM (2011). Melittin restores proteasome function in an animal model of ALS. *Journal of Neuroinflammation*.

[15] Rosen DR, Siddique T, Patterson D (1993). Mutations in Cu/Zn superoxide dismutase gene are associated with familial amyotrophic lateral sclerosis. *Nature*.

[16] Yin CS, Jeong HS, Park HJ (2008). A proposed transpositional acupoint system in a mouse and rat model. *Research in Veterinary Science*.

[17] Benner EJ, Banerjee R, Reynolds AD (2008). Nitrated *α*-synuclein immunity accelerates degeneration of nigral dopaminergic neurons. *PLoS ONE*.

[18] Stone DK, Reynolds AD, Mosley RL, Gendelman HE (2009). Innate and adaptive immunity for the pathobiology of Parkinson’s disease. *Antioxidants and Redox Signaling*.

[19] Frank-Cannon TC, Alto LT, McAlpine FE, Tansey MG (2009). Does neuroinflammation fan the flame in neurodegenerative diseases?. *Molecular Neurodegeneration*.

[20] Hensley K, Mhatre M, Mou S (2006). On the relation of oxidative stress to neuroinflammation: lessons learned from the G93A-SOD1 mouse model of amyotrophic lateral sclerosis. *Antioxidants and Redox Signaling*.

[21] Prudencio M, Hart PJ, Borchelt DR, Andersen PM (2009). Variation in aggregation propensities among ALS-associated variants of SOD1: correlation to human disease. *Human Molecular Genetics*.

[22] Chattopadhyay M, Valentine JS (2009). Aggregation of copper-zinc superoxide dismutase in familial and sporadic ALS. *Antioxidants and Redox Signaling*.

[23] Chen H, Jacobs E, Schwarzschild MA (2005). Nonsteroidal antiinflammatory drug use and the risk for Parkinson's disease. *Annals of Neurology*.

[24] Koprich JB, Reske-Nielsen C, Mithal P, Isacson O (2008). Neuroinflammation mediated by IL-1*β* increases susceptibility of dopamine neurons to degeneration in an animal model of Parkinson’s disease. *Journal of Neuroinflammation*.

[25] Chung CY, Koprich JB, Siddiqi H, Isacson O (2009). Dynamic changes in presynaptic and axonal transport proteins combined with striatal neuroinflammation precede dopaminergic neuronal loss in a rat model of AAV *α*-synucleinopathy. *Journal of Neuroscience*.

[26] Rott R, Szargel R, Haskin J (2008). Monoubiquitylation of *α*-Synuclein by seven in absentia homolog (SIAH) promotes its aggregation in dopaminergic cells. *Journal of Biological Chemistry*.

[27] Baba M, Nakajo S, Tu PH (1998). Aggregation of *α*-synuclein in Lewy bodies of sporadic Parkinson’s disease and dementia with Lewy bodies. *American Journal of Pathology*.

[28] Periquet M, Fulga T, Myllykangas L, Schlossmacher MG, Feany MB (2007). Aggregated *α*-synuclein mediates dopaminergic neurotoxicity in vivo. *Journal of Neuroscience*.

[29] Hanger DP, Brion JP, Gallo JM, Cairns NJ, Luthert PJ, Anderton BH (1991). Tau in Alzheimer’s disease and Down’s syndrome is insoluble and abnormally phosphorylated. *Biochemical Journal*.

[30] Mezey E, Dehejia A, Harta G, Papp MI, Polymeropoulos MH, Brownstein MJ (1998). Alpha synuclein in neurodegenerative disorders: murderer or accomplice?. *Nature Medicine*.

[31] Johnson WG (2000). Late-onset neurodegenerative diseases—the role of protein insolubility. *Journal of Anatomy*.

[32] Bence NF, Sampat RM, Kopito RR (2001). Impairment of the ubiquitin-proteasome system by protein aggregation. *Science*.

[33] Alves-Rodrigues A, Gregori L, Figueiredo-Pereira ME (1998). Ubiquitin, cellular inclusions and their role in neurodegeneration. *Trends in Neurosciences*.

[34] Bennett MC, Bishop JF, Leng Y, Chock PB, Chase TN, Mouradian MM (1999). Degradation of *α*-synuclein by proteasome. *Journal of Biological Chemistry*.

[35] Webb JL, Ravikumar B, Atkins J, Skepper JN, Rubinsztein DC (2003). *α*-Synuclein is degraded by both autophagy and the proteasome. *Journal of Biological Chemistry*.

[36] Zheng Q, Li J, Wang X (2009). Interplay between the ubiquitin-proteasome system and autophagy in proteinopathies. *International Journal of Physiology, Pathophysiology and Pharmacology*.

[37] Crippa V, Sau D, Rusmini P (2010). The small heat shock protein B8 (HspB8) promotes autophagic removal of misfolded proteins involved in amyotrophic lateral sclerosis (ALS). *Human Molecular Genetics*.

[38] Hara T, Nakamura K, Matsui M (2006). Suppression of basal autophagy in neural cells causes neurodegenerative disease in mice. *Nature*.

[39] Chen S, Zhang X, Song L, Le W (2012). Autophagy dysregulation in amyotrophic lateral sclerosis. *Brain Pathology*.

[40] Sasaki S (2011). Autophagy in spinal cord motor neurons in sporadic amyotrophic lateral sclerosis. *Journal of Neuropathology and Experimental Neurology*.

[41] Morimoto N, Nagai M, Ohta Y (2007). Increased autophagy in transgenic mice with a G93A mutant SOD1 gene. *Brain Research*.

[42] Hadano S, Otomo A, Kunita R (2010). Loss of ALS2/Alsin exacerbates motor dysfunction in a SOD1-expressing mouse ALS model by disturbing endolysosomal trafficking. *PloS One*.

